# Genetics and clinical phenotype of Erdheim–Chester disease: A case report of constrictive pericarditis and a systematic review of the literature

**DOI:** 10.3389/fcvm.2022.876294

**Published:** 2022-08-11

**Authors:** Lorenzo Bartoli, Francesco Angeli, Andrea Stefanizzi, Michele Fabrizio, Pasquale Paolisso, Luca Bergamaschi, Alessandro Broccoli, Pier Luigi Zinzani, Nazzareno Galiè, Paola Rucci, Alberto Foà, Carmine Pizzi

**Affiliations:** ^1^Institute of Cardiology, Sant’Orsola-Malpighi Hospital, IRCCS, Bologna, Italy; ^2^Department of Experimental, Diagnostic and Specialty Medicine-DIMES, University of Bologna, Bologna, Italy; ^3^Cardiovascular Center Aalst, OLV-Clinic, Aalst, Belgium; ^4^Department of Advanced Biomedical Sciences, University of Naples Federico II, Naples, Italy; ^5^Institute of Hematology “L. e A. Seràgnoli”, Sant’Orsola-Malpighi Hospital, IRCCS, Bologna, Italy; ^6^Division of Hygiene and Biostatistics, Department of Biomedical and Neuromotor Sciences, Alma Mater Studiorum, University of Bologna, Bologna, Italy

**Keywords:** Erdheim-Chester disease, constrictive pericarditis, genotype, phenotype, precision medicine, case-report

## Abstract

**Background:**

Erdheim–Chester disease (ECD) is a rare form of histiocytosis. An increasing number of genetic mutations have been associated with this syndrome, confirming its possible neoplastic origin. Recently, a connection between the BRAF mutational status and a specific phenotype was described; however, no studies have yet evaluated the correlations between other mutations and the clinical features of the disease.

**Objectives:**

This study aims to clarify the association between the clinical phenotype and genetic mutations identified in the neoplastic cell lines of ECD.

**Methods:**

We describe a case of ECD characterized by pericardial involvement and a KRAS mutation shared with chronic myelomonocytic leukemia. Hence, through a meta-analysis of individual participant data of all genetically and clinically described cases of ECD in the literature, we aimed to elucidate the association between its clinical phenotype and baseline genetic mutations.

**Results:**

Of the 760 studies screened, our review included 133 articles published from 2012 to April 2021. We identified 311 ECD patients whose genotype and phenotype were described. We found five main genes (BRAF, KRAS, NRAS, PIK3CA, and MAP2K1) whose mutation was reported at least three times. Mutation of BRAF led to a neurological disease (183 of 273 patients, 67%; *p* < 0.001); KRAS- and NRAS-mutated patients mainly showed cutaneous (five of six patients, 83.3%, *p* < 0.004) and pleural (four of nine patients, 44%, *p* = 0.002) involvement, respectively; PIK3CA was not associated with specific organ involvement; and MAP2K1 mutations caused the disease to primarily involve the peritoneum and retroperitoneum (4 of 11, 36.4%, *p* = 0.01).

**Conclusion:**

This work implies a possible influence of baseline mutation over the natural history of ECD, underscoring the importance of a thorough genetic analysis in all cases with the ultimate goal of identifying a possible targeted therapy for each patient.

## Introduction

Erdheim–Chester disease (ECD) is a rare non-Langerhans cell histiocytosis (1, 2), with less than 1,000 cases described so far (2). It is known that ECD affects multiple organs with a wide spectrum of clinical presentations, ranging from an asymptomatic to a life-threatening condition. The most common symptom is bone pain (50%) as bilateral symmetric osteosclerosis evaluated by 18F-fluorodeoxyglucose (18F-FDG) positron emission tomography/computed tomography (PET/CT) or technetium-99 m (Tc-99m) bone scan is considered a pathognomonic feature, reported by about 80–95% of patients (3, 4). The other organs often infiltrated are the skin (xanthelasmas), central nervous system (CNS; resulting in ataxia, dysarthria, cognitive impairment, headache, peripheral neuropathy, and, sometimes, mood lability), respiratory system, pituitary gland (mostly central diabetes insipidus), retroperitoneum (renal failure requiring dialysis), and retro-orbital tissue (exophthalmos, retro-orbital pain, oculomotor nerve palsy, or vision loss; 2–4). About 50–70% of patients with ECD exhibit cardiovascular involvement. The most common findings include pericardial infiltration and effusion, and right atrioventricular pseudotumor and peri-arterial sheathing, involving mainly the thoracic and abdominal aorta, renal vessels, and coronary arteries (2–4).

Supplementary Box 1 summarizes the multiorgan involvement in ECD and the potential “red flags.” An increasing number of genetic mutations have been associated with ECD. Specifically, recurrent activating mutations involving the mitogen-activated protein kinase and phosphatidylinositol 3-kinase (PI3K)-AKT pathways have been discovered. BRAF V600E is the most frequent mutation encountered (5), although other genes may be affected, such as NRAS, KRAS, PI3KCA, ARAF, MAP2K1, ALK, and NTRK1 (6–9).

These findings suggest that ECD is a clonal neoplastic disorder, and the most common signaling pathways involved could represent the first molecular targets in histiocytic disorders (7, 8, 10–12).

Last, an association between ECD and leukemic/myelodysplastic disorders that share most of the clonal mutations has been described (13), confirming the possible neoplastic origin probably due to a clonal hematopoiesis phenomenon (14–16).

In terms of clinical presentation, an association between the BRAF mutational status and cardiac, as well as neurological, disorders has been reported. Nevertheless, no studies have yet evaluated the correlations between other gene mutations and the specific ECD phenotype.

We report a case of constrictive pericarditis in a man with xanthelasmas and monocytosis; the final diagnosis was ECD associated with chronic myelomonocytic leukemia (CMML) harboring the same clonal mutation in the KRAS gene. Furthermore, we report the results of a meta-analysis of individual participant data (IPD) of all available ECD cases, aiming to elucidate the association between the ECD clinical phenotype and genetic mutations in neoplastic cell lines.

## Case report

**FIGURE 1:**
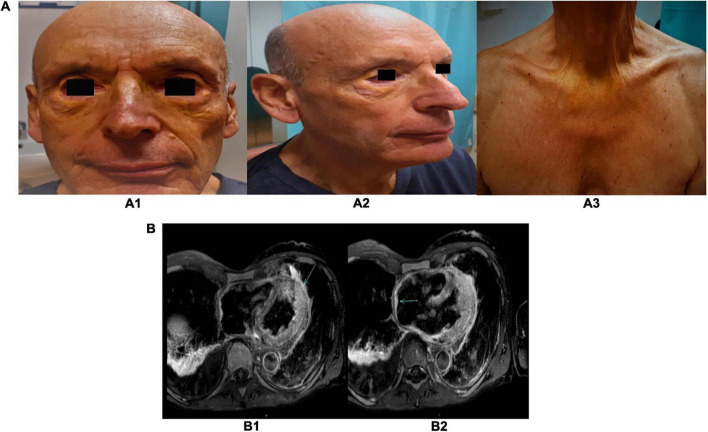
**(A)** Pictures of the xanthelasmas of the eyelid and thorax. Several yellowish plaque/papules on both upper and lower eyelids, cheeks **(A1,A2)**, and anterior region of the thorax **(A3)**. **(B)** Cardiac magnetic resonance images. Post-gadolinium T2-weighted sequences, showing a gadolinium-enhanced pericardial nodule **(B1)** and the pseudotumor of the right atrial roof **(B2)**.

In the early 2000s, a 55-year-old man developed bilateral eyelid and thorax xanthelasmas (Figure 1A). The eyelid lesions relapsed rapidly after two surgical excisions.

In 2011, blood tests revealed monocytosis, and in the following years, splenomegaly was detected. No further diagnostic or therapeutic approaches were applied due to the lack of systemic and specific organ symptoms, as well as blood test alterations.

In 2017, the patient underwent left pleurodesis due to left pleuritis after a series of ineffective conservative treatments. Bioptic and cytologic materials revealed non-specific inflammation.

The patient remained asymptomatic until October 2019, when he developed fatigue and breathlessness. An electrocardiogram demonstrated sinus tachycardia. Right pleural effusion was detected on chest X-ray. Echocardiography showed findings consistent with constrictive pericarditis; therefore, he was admitted to our cardiology unit.

Blood tests confirmed monocytosis with dysmorphic, broken, and vacuolated monocytes on a blood smear. In addition, anemia and elevated C-reactive protein were observed. Notably, despite the history of xanthelasmas, cholesterol levels were low without lipid-lowering therapy.

A chest CT scan detected nodular pleuro-pericardial lesions. Right heart catheterization and cardiac magnetic resonance (MR; Supplementary Video 1) confirmed the diagnosis of constrictive pericarditis. Furthermore, cardiac MR revealed right atrial posterior wall and pericardial thickening. It also confirmed nodular pericardial and pleural lesions showing hyperemia and edema in T2-weighted sequences (Figure 1B).

Given the multiorgan involvement, including the right atrium and pericardium, which are reported to be commonly affected in cardiac malignancies (17), we hypothesized a hematologic systemic disease. Specifically, the presence of xanthelasma, pleuro-pericardial involvement, and right atrial posterior wall thickening raised the suspicion of ECD.

## Methods

To investigate the possible bone involvement, we performed a bilateral femoral X-ray and MR scan.

Subsequently, a whole-body 99mTc-3,3-diphosphono-1,2-propanodicarboxylic acid (99mTc-DPD) bone scintigraphy and 18F-FDG PET/TC were performed to evaluate disease extension and to guide a subsequent biopsy.

We performed blood tests to exclude endocrinopathy, namely, adrenal and pituitary involvement. The CNS was evaluated by a head MR scan. To demonstrate pulmonary, perivascular, and perinephric infiltrates, CT scans of the neck, chest, abdomen, and pelvis were acquired.

The presence of peripheral monocytes and splenomegaly led us to perform a bone marrow biopsy. To detect the typical components of histiocytic infiltrates, tissue was taken from the xanthelasmas of upper lateral and lower medial left eyelids, and a bone biopsy from regions with maximum osteosclerosis on the previous femoral MR.

Moreover, immunohistochemistry (IHC) was performed to evaluate the presence of the typical BRAF V600E mutation. Targeted capture next-generation sequencing (NGS) was executed to identify ECD-related mutations in the skin biopsy, blood plasma, and bone marrow. The panels used for genetic analysis, the genes specifically evaluated by these techniques, and methods used are reported in the section “Supplementary material.”

Last, we performed a meta-analysis of IPD of all ECD cases in the literature. Design and reporting were carried out according to PRISMA-IPD Statement (18) (Supplementary Tables 1, 2). Our methods are reported in the Supplementary material.

## Results

Bilateral femoral X-ray and MR revealed osteosclerosis. In addition, MR images showed a symmetrical increase in the red bone marrow. On the other hand, both whole-body 99mTc-DPD scintigraphy and 18F- FDG PET/CT were negative for bone tracer uptake. 18F-FDG PET/CT showed bone marrow glucose uptake consistent with a myeloproliferative disease and there was a mild uptake by the known pleural and pericardial nodular lesions (19).

The head MR scan excluded CNS, pituitary, or retro-orbital involvement. The CT scans of the neck, chest, abdomen, and pelvis only showed marked splenomegaly, without any further sign of organ involvement. The hypothalamic–pituitary–adrenal axis and thyroid and sex hormones were within the normal range.

A right femur biopsy did not show any relevant histiocytic and/or inflammatory component. Conversely, the skin specimen presented diffuse histiocytic infiltrates, with histopathologic and immunohistochemical features, consistent with ECD (CD 68+, CD163+, S100-, and CD1a-; Supplementary Figures 1A, A1, A2, and A3).

The bone marrow biopsy showed a monocytic component (CD14+, CD68PGM1+, and CD163+) compatible with CMML, a rare myeloproliferative disorder (Supplementary Figures 1B, B1, B2, and B3).

Next-generation sequencing analysis of the skin biopsy, blood, and bone marrow, and immunofluorescence of the skin biopsy excluded the presence of typical BRAFV600E mutation but showed a clonal mutation involving KRAS gene (p. Gly12Asp, c.35G > A9), shared by both histiocytes and leukemic cells (Supplementary Figures 1A, A4, 1B, B4). Moreover, adjunctive mutations were reported on the peripheral blood and bone marrow, namely, ASXL1 (p. Leu815Pro), SETBP1 (p.Val1101Ile a), EZH2 (p.Asp185His), and TP53 (p.Pro72Arg).

The final diagnosis was ECD and concomitant chronic myelomonocytic leukemia with a shared clonal mutation.

### Treatment and follow-up

A treatment with corticosteroid was started, and clinical conditions rapidly improved. A chest X-ray at 3 months showed the resolution of pleural effusion. Blood tests indicated a progressive increase in the blood cell count with no signs of inflammation markers. Cutaneous lesions showed a progressive reduction, leading to complete disappearance.

At the 6-month follow-up, the patient was completely asymptomatic. A cardiac MR scan demonstrated resolution of pericardial constriction (Supplementary Video 1), and neither hyperemia nor edema was observed at T2-weighted images. Similarly, a contrast-enhanced chest CT scan demonstrated complete resolution of pleuro-pericardial effusion and nodular lesions.

### Meta-analysis of individual participant data

**FIGURE 2:**
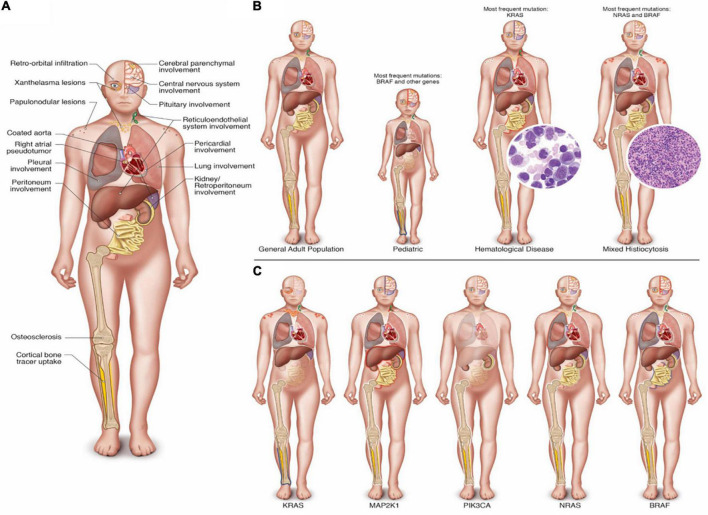
(Central Illustration): **(A)** Main clinical and radiographic findings in ECD. **(B)** Clinical and radiographic findings in specific subgroups of ECD. Characteristics of the general adult population, the pediatric population, patients with associated hematologic disorder, in mixed histiocytosis and the respective most frequently associated genetic mutations reported in the literature. **(C)** Clinical and radiographic findings of ECD in patients with mutation of KRAS, MAP2K1, PIK3CA, NRAS, and BRAF. Note: In **(B,C)**, clinical and radiological features of the specific subgroups were depicted transparent if they were never reported in the literature. Instead, they were reported with a blue halo if there was a statistically significant low frequency involvement; a white halo if there was a normal frequency involvement; and finally with a red halo if there was a statistically significant common involvement in the respective group.

Of 760 studies screened, our review of the literature selected 133 articles. The first article included was published in September 2012, and it was the first molecularly characterized case report of ECD (20). In the end, we identified 311 ECD patients whose genotype and phenotype were described (flow diagram of IPD search). The major traits of ECD are summarized in Figure 2, which illustrates the main clinical and radiographic findings of ECD (Figure 2A), the clinical features of specific subgroups (Figure 2B), and the influences of the main mutation over clinical phenotypes (Figure 2C).

We identified five main genes (BRAF, KRAS, NRAS, PIK3CA, and MAP2K1) whose mutation was reported at least three times in the literature. We merged all the others (Kit, JAK2, PDGFRA, ARAF, CSF1R, and FLT3-ITD) in the same group “other genes.”

BRAF was the most frequently mutated gene (Table 1). Its mutation is linked to neurological disease (67.9%), with one-third of the patients showing pituitary involvement, while in less than 10%, the pleura and peritoneum were affected (Tables 2, 3, and Figure 2C).

**TABLE 1:** General characteristics of Erdheim–Chester patients according to different genotypes.

	NRAS	KRAS	BRAF	MAP2K1	PIK3CA	Other	*P* value
		
	*n* = 9	*n* = 6	*n* = 273	*n* = 11	*n* = 3	*n* = 8	
Male gender, *n* (%)	8 (88.9)	2 (33.3)	172 (65.6)	10 (90.9)	3 (100)	6 (75.0)	ns
Age, mean ± SD	59.9 ± 9.8	59.8 ± 9.4	52.2 ± 17.7	51.9 ± 11.6	31 ± 1.7	43.6 ± 28.1	ns
Pediatric patient, *n* (%)	0 (0.0)	0 (0.0)	16 (5.9)	0 (0.0)	0 (0.0)	2 (25.0)	ns
Multigene involvement, *n* (%)	0 (0.0)	3 (50) *	14 (5.1)	1 (9.1)	0 (0.0)	4 (50.0) *	**0.001**
Concomitant myeloid	3	4	26	1	0	2	**0.001**
or lymphoid neoplasms, *n* (%)	(33.3)	(66.7) *	(9.5)	(9.1)	0	(25)	
**Type of concomitant neoplasm, *n* (%)**							
Acute	1 (33.3)	0 (0.0)	6 (23.1)	0 (0.0)	0 (0.0)	1 (50.0)	ns
Chronic	3 (100.0)	4 (100.0)	22 (84.6)	1 (100.0)	0 (0.0)	1 (50.0)	ns
Lymphoid	0 (0.0)	0 (0.0)	3 (11.5)	0 (0.0)	0 (0.0)	0 (0.0)	ns
Myeloid	3 (100.0)	4 (100.0)	19 (73.1)	1 (0.0)	0 (0.0)	2 (100.0)	ns
Myelodysplasia	0 (0.0)	0 (0.0)	4 (15.4)	0 (0.0)	0 (0.0)	0 (0.0)	ns
Mixed Histiocytosis	1 (11.1)	0 (0.0)	24 (8.8)	1 (9.1)	0 (0.0)	0 (0.0)	ns
Shared mutation between ECD and neoplasm	3 (100.0) *	4 (100.0) *	1 (7.7)	0 (0.0)	0 (0.0)	2 (100.0) *	**0.004**

*Categories for which there is a statistically significant difference. Statistically significant p-values are in bold.

**TABLE 2:** Clinical characteristics of Erdheim–Chester patients according to different genotypes.

	NRAS	KRAS	BRAF	MAP2K1	PIK3CA	Other	*P* value
		
	*n* = 9	*n* = 6	*n* = 273	*n* = 11	*n* = 3	*n* = 8	
Cardiovascular involvement, *n* (%)	5	3	131	6	1	2	ns
	(55.6)	(50)	(47.8)	(54.5)	(33.3)	(25)	
Central nervous system involvement, *n* (%)	3	0	183	5	1	2	**<0.001**
	(33.3)	0	(67) *	(45.5)	(33.3)	(25)	
Bone involvement, *n*(%)	6	4	232	10	3	6	ns
	(66.7)	(66.7)	(85)	(90.9)	(100)	(75)	
Cortical bone tracer uptake, *n*(%)	3	1	161	9	3	5	**0.001**
	(100)	(33.3) *	(94.7)	(90)	(100)	(100)	
Lung involvement, *n* (%)	4	2	62	1	0	3	ns
	(44.4)	(33.3)	(22.7)	(9.1)	(0.0)	(37.5)	
Kidney/retroperitoneum involvement, *n* (%)	6	0	155	10	1	4	**0.009**
	(66.7)	0	(56.7)	(90.9) *	(33.3)	(50)	
Skin involvement, *n* (%)	3	5	77	3	0	4	**0.004**
	(33.3)	(83.3) *	(28.2)	(27.3)	(0.0)	(50)	
Serosal involvement, *n* (%)	5	3	79	4	0	3	ns
	(55.6)	(50)	(28.9)	(36.4)	(0.0)	(37.5)	
Reticuloendothelial system involvement, *n* (%)	1	1	14	3	0	2	**0.01**
	(11.1)	(16,6)	(5.1)	(27.3) *	(0.0)	(25)	

*Categories for which there is a statistically significant difference. Statistically significant p-values are in bold.

**TABLE 3:** Specific cardiac and non-cardiac involvements of Erdheim–Chester patients according to different genotypes.

	NRAS	KRAS	BRAF	MAP2K1	PIK3CA	Other	*P* value
		
	*n* = 9	*n* = 6	*n* = 273	*n* = 11	*n* = 3	*n* = 8	
Coated aorta, *n* (%)	5	0	84	5	1	1	ns
	(55.6)	(0.0)	(32.3)	(45.5)	(33.3)	(12.5)	
Peri-myocardial formations^a^, *n* (%)	1	1	65	1	0	1	ns
	(11.1)	(16.7)	(24.6)	(9.1)	(0.0)	(12.5)	
Pericardial involvement, *n* (%)	0	2	51	1	0	2	ns
	(0.0)	(33.3)	(19.6)	(9.1)	(0.0)	(25.0)	
Cerebral parenchymal involvement, *n* (%)	2	0	115	1	1	0	**0.008**
	(22.2)	(0.0)	(42.1) *	(9.1) *	(33.3)	(0.0)	
Pituitary involvement, *n* (%)	0	0	91	3	0	0	**0.009**
	(0.0)	(0.0)	(33.3) *	(27.3)	(0.0)	(0.0)	
Retro-orbital infiltration, *n* (%)	1	0	52	1	1	2	ns
	(11.1)	(0.0)	(19)	(9.1)	(33.3)	(25.0)	
Pleural involvement, *n* (%)	4	2	25	1	0	3	**0.002**
	(44.4) *	(33.3)	(9.2) *	(9.1)	(0.0)	(37.5)	
Peritoneal involvement, *n* (%)	2	0	24	4	0	2	**0.01**
	(22.2)	(0.0)	(8.8) *	(8.8) *	(0.0)	(25.0)	
Xanthelasma lesions, *n* (%)	3	3	38	0	0	4	**0.005**
	(33.3)	(50.0) *	(14.6)	(0.0)	(0.0)	(50.0) *	
Papulonodular lesions, *n* (%)	0	3	24	2	0	1	**0.01**
	(0.0)	(50.0) *	(9.2)	(18.2)	(0.0)	(12.5)	

^a^Presence of at least one between myocardial, right atrium and atrio-ventricular junction infiltration.

*Categories for which there is a statistically significant difference. Statistically significant p-values are in bold.

KRAS mutation is closely related to the skin (both xanthelasma-like and nodular lesions). Furthermore, there was a lower incidence of bone tracer uptake at PET-FDG and bone scan (in case of bone involvement) and a higher frequency of concomitant hematologic neoplasia. The CNS, retroperitoneum, and mesentery were never affected. Finally, patients with KRAS-mutated ECD frequently showed multigene involvement (Tables 1–3 and Figure 2C). These features were also observed in our case report.

Erdheim–Chester disease patients carrying NRAS mutations did not present pericardial or pituitary disease but had a high frequency of pleural involvement (Tables 2, 3, and Figure 2C).

Patients with MAP2K1-mutated ECD showed more frequent abdominal involvement, with both peritoneal and retroperitoneal fibrosis and/or hairy kidney features. Moreover, they tended to show reticuloendothelial system involvement, and they never had xanthelasma lesions (Tables 2, 3, and Figure 2C).

PIK3CA mutation affected young patients without a specific organ involvement. Notably, the lung, skin, serosa, pituitary gland, and reticuloendothelial system were never affected (Tables 1–3 and Figure 2C).

Furthermore, we noticed peculiar characteristics of three different populations: pediatric individuals, patients with concomitant hematologic disease, and subjects with mixed histiocytosis (defined as the simultaneous presence of another histiocytic disease).

In pediatric patients, the most frequently mutated gene was BRAF. These patients frequently exhibited CNS involvement with a lower incidence of serosal and bone disease (with lower tracer uptake at PET-FDG or bone scan), while cardiovascular involvement and abdominal involvement were never observed (Supplementary Table 3 and Figure 2B).

Patients with an associated hematologic neoplasia are older, with a higher frequency of pulmonary, reticuloendothelial, and serosal manifestations, while CNS involvement is sporadic. Notably, subjects with KRAS or NRAS-mutated ECD frequently shared the genetic mutation between histiocytes and hematologic neoplasia (Supplementary Table 4 and Figure 2B).

Finally, patients with mixed histiocytosis showed clinical features similar to those of the conventional ECD cohort with, a higher incidence of skin involvement, such as papulonodular lesions (Supplementary Table 5 and Figure 2B).

## Discussion

Our clinical case demonstrated that targeted therapy is driven by a precise diagnosis.

The goal of precision medicine is “to provide the best available care for each individual,” (21) and an accurate diagnostic approach is crucial to allow the best therapeutic option. This concept is pivotal in entities with a heterogeneous clinical presentation, such as ECD.

In fact, our clinical case differs from the classical presentation of this disease. The patient developed bilateral eyelid and thorax xanthelasmas, pleuritis, and constrictive pericarditis without typical ECD findings, such as tracer uptake on the bone scan or FDG-PET, CNS involvement, coated aorta, and the hairy kidney/retroperitoneal fibrosis, which usually raise the suspicion of ECD.

Furthermore, the hematologic neoplasm and the presence of histiocytes and leukemic cells of KRAS mutation were confounding factors in the diagnostic process.

Precision medicine means to match clinical, diagnostic, and genetic features of a disease. Therefore, to further promote the use of precision medicine and considering the growing body of evidence that ECD is a clonal hematopoietic disorder (3), we hypothesized that extremely variable clinical features may relate to the primitive mutations that generate neoplastic germlines. These germlines give birth to both histiocytic and, if present, hematologic neoplastic cells, and the baseline genetic mutations could greatly influence the clinical phenotype and the natural history of the disease.

To test this hypothesis, we performed an individual meta-analysis of all case reports and case series of patients with a diagnosis of ECD and known mutational status. The results of our work, which is the most comprehensive to date, support our hypothesis since each mutation demonstrated a trend toward organ-specific involvement.

Not surprisingly, the clinical features of our case matched with the results of our analysis since they could be explained by the KRAS mutated status and the concomitant hematologic neoplasm.

Our results, which help in the diagnostic process and promote a thorough genetic analysis of ECD, may have also a positive therapeutic and prognostic impact following a precision model of therapy for Mendelian disease, focused on treating the underlying mechanism using a genetic therapy.

This approach is increasingly used and continuously developing: BRAF and MEK/ERK inhibitors have been already used extensively in ECD (7, 8, 10–12), while others have been used in sporadic case reports. Moreover, a new molecule targeting KRAS has been successfully administrated for the first time in a phase I trial in lung cancer (4, 22) and has recently been approved by the Food and Drug Administration for therapeutic use (23). Our work suggests a possible influence of baseline mutation over the natural history of the disease, underscoring the importance of a thorough genetic analysis in all ECD cases with the ultimate goal of identifying a targeted therapy for each patient.

## Study limitations

Our sample size is small (311 patients), except for BRAF, which had a small number of cases for other mutations. This is due to the rarity of this syndrome. Also, there is lack of systematic or thorough genetic analysis in all cases found in the literature, so a high number of patients could not be included in our study owing to the absence of a known mutation.

On the contrary, some studies were excluded because they did not provide the clinical phenotype of the study population, or because a clear link between the genotype and phenotype of individual patients was not always possible (i.e., aggregate data).

Since our meta-analysis focused only on those cases whose both genotype and clinical phenotype were available, confounding and selection bias cannot be excluded.

Finally, there was a risk of including low-quality articles or studies with heterogenous data. Nevertheless, data from individual patients retrieved from different articles were harmonized for statistical analysis, and the methodological quality of the studies was assessed using the method proposed by Murad et al. (see Supplementary material). Thus, of the 131 studies included, 106 and 27 showed good and moderate overall quality, respectively. No study revealed a low methodological quality.

## Data availability statement

The original contributions presented in the study are included in the article/Supplementary material, further inquiries can be directed to the corresponding authors.

## Ethics statement

Written informed consent was obtained from the individual(s) for the publication of any potentially identifiable images or data included in this article. Written informed consent was obtained from the participant/s for the publication of this case report.

## Author contributions

LBa and FA contributed to the conception or design of the work and drafted the manuscript. AS, MF, PP, LBe, and PR contributed to the acquisition, analysis, or interpretation of data for the work. AB, PZ, NG, AF, and CP critically revised the manuscript. All authors gave final approval and agreed to be accountable for all aspects of work ensuring integrity and accuracy.

## Supplementary material

Supplementary Figure 1 **(A)** Biopsy of the eyelid. Images of hematoxylin-eosin stain **(A1)**, CD 68 **(A2)**, and CD163 **(A3)** immunohistochemistry. The illustrations show foamy, CD68+ and CD163+ histiocytes, typical of Erdheim-Chester disease. In the last image **(A4)**, demonstration of a KRAS exon 2 mutation (p.Gly12Asp) by NGS (S5 Gene Studio) in the specimen. **(B)** Biopsy of the bone marrow (BM). Images of hematoxylin-eosin **(B1)**, and May–Grünwald **(B2)** stain and CD14 immunohistochemistry **(B3)**. The illustrations show granulocytic proliferation in an hypercellular bone marrow, with monocytes (CD14+), erythroid precursors and abundant hypolobulated and small megakaryocytes, suggestive of chronic myelomonocytic leukemia. The last image **(B4)** shows the detection by NGS (S5 Gene Studio) in both BM and peripheral blood of the same KRAS mutation already detected in the eyelid specimen.

Supplementary Video 1 In the video frames real T1 cine sequences showing evident respiratory shift of the interventricular septum due to enhanced ventricular interdependence, which completely resolves at follow-up.
