# Supplementary material for: Genetics and clinical phenotype of Erdheim–Chester disease: A case report of constrictive pericarditis and a systematic review of the literature
Source: Front Cardiovasc Med. 2022 Aug 11;9:876294. doi: 10.3389/fcvm.2022.876294 (PMC9403274; doi:10.3389/fcvm.2022.876294)
Supplement: Supplementary file 1 [file Data_Sheet_1.pdf]

SUPPLEMENTARY MATERIALS

METHODS FOR THE DIAGNOSTIC PATHWAY:

Whole-body 99mTc-3,3-diphosphono-1,2-propanodicarboxylic acid (99mTc-DPD) bone scintigraphy and 18F-fluorodeoxyglucose positron emission tomography-computed tomography (18F-FDG PET-CT) were performed according to the European association of nuclear medicine (EANM) guidelines1,2.

Bone marrow biopsy was performed using a Jamshidi needle to obtain material from the right posterior iliac crest in local anesthesia. The bone biopsy was performed using a hybrid echographic and fluoroscopic guide.

Tissue from the bone, bone marrow and skin biopsies was formalin fixed and colored with hematoxylin and eosin (Supplementary Figure, Panel A, A1). We then used immunohistochemistry (IHC) to assess the typical immunophenotype of ECD cells. (Supplementary Figure, Panel A, A2 and A3).

Targeted-capture next-generation sequencing (NGS) was executed to look for Erdheim Chester disease (ECD)-related mutations. Specifically, we explored the mutational status of selected regions within the main 54 genes involved in tumorigenesis of the skin biopsy with “Oncomine Focus Assay” panel, while we performed an analysis of 25 genes on bone marrow and cell-free DNA (cfDNA) plasma with “Myeloid Solution panel”.

We used the Ion Chef to prepare libraries for emulsion polymerase-chain reaction (em-PCR) and chip loading, while sequencing was done on the Ion S5 GeneStudio. Then, we performed data analysis with Ion Reporter v.5.10 software.

PANELS USED FOR GENETIC CHARACTERIZATION IN OUR CASE REPORT

For the skin biopsy, we used Oncomine focus assay that evaluates a panel of 35 genes, including AKT1, ALK, AR, BRAF, CDK4, CTNNB1, DDR2, EGFR, ERBB2, ERBB3, ERBB4, ESR1, FGFR2, FGFR3, GNA11, GNAQ, HRAS, IDH1, IDH2, JAK1, JAK2, JAK3, KIT, KRAS, MAP2K1,

MAP2K2, MET, MTOR, NRAS, PDGFRA, PIK3CA, RAF1, RET, ROS1 e SMO), and amplifies 19 genes (ALK, AR, BRAF, CCND1, CDK4, CDK6, EGFR, ERBB2, FGFR1, FGFR2, FGFR3, FGFR4, KIT, KRAS, MET, MYC, MYCN, PDGFRA e PIK3CA).

Moreover, we used Myeloid solution panel for blood and bone marrow genetic analysis. This evaluated a total of 25 genes including ABL1, ASXL1, BRAF, CALR, CBL, CEBPa, CSF3R, DNMT3A, ETV6, EZH2, FLT3, HRAS, IDH1, IDH2, JAK2, KIT, KRAS, MPL, NPM1, NRAS, PTPN11, RUNX1, SETBP1, SF3B1, SRSF2, TET2, TP53, U2AF1 (2, 6), WT1 (6-10), ZRSR2.

META-ANALYSIS OF INDIVIDUAL PARTICIPANT DATA:

Search, and study/patient inclusion criteria:

We analyzed all the case series or case reports published from 2010 to April 2021 on MedLine, Scopus, EMBASE, and the Cochrane Controlled Clinical Trial Register. The bibliographies of all relevant articles were reviewed.

The search string was adjusted for each database while maintaining a common overall architecture. We used the following terms: “Erdheim” OR “Erdheim-Chester” OR “Chester” OR “Erdheim Chester” or “histiocytosis”. Further, we used various combinations of the following terms related to two main domains: “Erdheim” OR “Erdheim-Chester” OR “Chester” OR “Erdheim Chester” or “histiocytosis” AND “genetics” OR “mutation” OR “genotype” OR “leukemia” OR “lymphoma” OR “neoplasia” OR “mixed histiocytosis” OR “Langerhans Cell Histiocytosis” OR “Rosai-Dorfman” OR “BRAFV600E“ OR “BRAF” OR “NRAS” OR “KRAS” OR “PI3KCA” OR “ARAF” OR “MAP2K1”

Study inclusion criteria were: (a) patients with a definitive diagnosis of ECD (b) patients whose genotype and phenotype features were available.

We included articles written in English, German, Italian and Spanish. Among articles published by the same Institution, we included only those patients which could be individually distinguished and reporting only the latest information, thus avoiding redundancies (number of articles excluded=7).

We excluded duplicates (n=22), articles with lack of patients' genotype and/or phenotype (n=583) and studies for which IPD were not provided (patients' genotype and/or phenotype not completely described, or patients' genotype and phenotype not clearly associated, n=15). We included articles in which BRAF mutational status was assessed by immunofluorescence.

For each patient, we collected data regarding age, sex, genetic mutations, major comorbidities (concomitant hematological neoplasms or histiocytosis) and main clinical or radiological disease involvement. When available, information related to therapy and follow-up was gathered.

Finally, 15 individual participants were excluded from our analysis

The patient reported in our article was included in the meta-analysis.

Data extraction:

Using a standardized approach, 2 independent investigators (L.B. and F.A.) extracted all data and inserted them into a common database. Discrepancies were resolved through common revision of the articles and group consultations. The extracted information included: editorial information (lead author, publication year, study institution, study design, and publication status), clinical presentation (patient age and sex, cardiovascular - pericardial disease, coated aorta, myocardial, right atrium and atrio-ventricular junction infiltration – bone - bone lesions and presence of bone tracer uptake at bone scan or 18F-fluorodeoxyglucose (18F-FDG) positron emission tomography/computed tomography (PET/CT) – central nervous system– parenchymal or dural lesions, pituitary involvement, retro-orbital disease – lung – pleural or parenchymal disease – abdominal – peritoneal inflammation, hairy kidney and retroperitoneal fibrosis – skin – xanthelasma, papulonodular or other lesions – and peculiar – adrenal, spleen, lymph nodes, hepatic, testes and upper respiratory way lesions - involvement) genotype (main identified gene, additional single gene or complex mutations), concomitant histiocytosis or concomitant hematological neoplasia (presence and genetic features - main identified gene, additional single gene or complex mutations, shared mutational status with Erdheim-Chester Disease, ECD).

Quality assessment data:

Since all the retrieved studies were case reports or case series, we assessed the aspects of the reported methodological quality using the "Tool for evaluating the methodological quality of case reports and case series" proposed by Murad et al. The latter assesses the methodological quality of the studies focusing on the following standardized tools:

a: Selection: 1. Does the patient(s) represent(s) the whole experience of the investigator (center) or is the selection method unclear to the extent that other patients with similar presentation may not have been reported?

b: Ascertainment: 2. Was the exposure adequately ascertained? 3. Was the outcome adequately ascertained?

c: Causality: 4. Were other alternative causes that may explain the observation ruled out? 5. Was there a challenge/rechallenge phenomenon? 6. Was there a dose–response effect? 7. Was follow-up long enough for outcomes to occur?

d: Reporting: 8. Is the case(s) described with sufficient details to allow other investigators to replicate the research or to allow practitioners make inferences related to their own practice?

We modified the abovementioned tools to underscore the importance of some of the elements presented in the articles. Therefore, we eliminated question 5-7 since they seemed redundant or unnecessary to our evaluation. The most important point was ascertainment, since our article focused on genotype and phenotype (the exposure and the outcome respectively). So, the ascertainment item had a major influence on the overall judgment, while all other items were equally evaluated.

According to Murad et al, we did not to create a numerical score; instead, we gave an overall judgment distinguishing good, moderate, and low-quality case series/case reports. Consequently, the item ascertainment was considered as double value. The overall judgment resulted in defining good quality level the presence of at least three items of good quality if the other two items were of moderate

quality, instead, if one of the two articles were of low-quality level, the article was considered of moderate quality. The remaining cases was classified as low-quality.

Statistical analysis:

Data from individual patients retrieved from different published studies were harmonized for the statistical analysis.

The association between demographic characteristic, the primary gene and the system involved was investigated using continency tables and chi-square test for categorical variables and one-way ANOVA for continuous variables.

The significance level was set to $p<0.01$.

SUPPLEMENTARY MATERIAL REFERENCES:

1. Jamar F, Buscombe J, Chiti A, Christian PE, Delbeke D, Donohoe KJ, Israel O, Martin-Comin J, Signore A. EANM/SNMMI guideline for 18F-FDG use in inflammation and infection. J Nucl Med. 2013 Apr;54(4):647-58.
2. Van den Wyngaert, T., Strobel, K., Kampen, et al. The EANM practice guidelines for bone scintigraphy. Eur J Nucl Med Mol Imaging 43, 1723–1738 (2016).

FLOW DIAGRAM OF INDIVIDUAL PATIENT DATA (IPD) SEARCH:

Number of studies identified through database searching = 760

↓

Number of studies after duplicates removed = 738

↓

Number of studies screened for eligibility = 738 → Number of studies excluded (lack of patients' genotype and/or phenotype) = 583

↓

Number of studies for which IPD were sought = 155 → Number of eligible Studies for which IPD were not sought (articles published by the same Institution*) = 7

↓

Number of studies for which IPD were provided = 133

Number of participants for whom data were provided = 311

Number of participants for whom no data were provided (patients' genotype and/or phenotype not available or not completely described, or absence of association between genotype and phenotype of each patient) = 15

Number of studies for which IPD were not provided (patients' genotype and/or phenotype not completely described, or patients' genotype and phenotype not clearly associated (i.e aggregate data)) = 15

Number of participants = 37

↓

IPD (report for each main outcome)

Number of studies included in analysis = 133

Number of studies excluded = 627

Number of participants included in analysis = 311

Number of participants excluded = 52

* When an Institution published two or more case-report/case series, we included only the latest studies and those where individual patients could be distinguished, thus avoiding redundancies.

SUPPLEMENTARY TABLES

Table 3. Features of pediatric patients.

	**Pediatric patients**<br>**n= 18**	**Non pediatric patients**<br>**n = 292**	**P value**
Cardiovascular involvement, n (%)	0<br>(0.0)	141<br>(48.3)	**<0.001**
Central nervous system involvement, n (%)	15<br>(83.3)	180<br>(61.6)	ns
Cerebral parenchymal involvement, n (%)	12<br>(66.7)	107<br>(36.6)	**0.01**
Pituitary involvement,<br>n (%)	9<br>(50)	80<br>(27.4)	ns
Bone involvement,<br>n (%)	9<br>(50.0)	252<br>(86,3)	**<0.001**
Cortical bone tracer uptake, n (%)	4<br>(57.1)	181<br>(95.2)	**<0.001**
Lung involvement,<br>n (%)	2<br>(11.1)	70<br>(24)	ns
Kidney/retroperitoneum involvement, n (%)	0<br>(0.0)	176<br>(61.0)	**<0.001**
Skin involvement,<br>n (%)	5<br>(27.8)	87<br>(29.8)	ns
Serosal involvement, n (%)	1<br>(5.6)	93<br>(31.8)	**0.009**
Pleural involvement, n (%)	1<br>(5.6)	34<br>(11,4)	ns
Reticuloendothelial system involvement, n (%)	1<br>(5.6)	19<br>(6.5)	ns
Mixed Histiocytosis, n (%)	3<br>(16.7)	23<br>(7.9)	ns
Concomitant myeloid or lymphoid neoplasms, n (%)	0<br>(0.0)	36<br>(12.3)	ns

Statistically significant p values are in bold

Table 4. Features of patients with associated myeloid/lymphoid neoplasia

	**Associated neoplasia**<br>**n= 36**	**Patients without associated neoplasia**<br>**n = 274**	**P value**
Age, mean ± SD	63<br>± 12	51.9<br>± 17.9	**<0.001**
Cardiovascular involvement, n (%)	22<br>(61.1)	126<br>(46)	ns
Central nervous system involvement, n (%)	15<br>(41.7)	180<br>(65.7)	**0.004**
Bone involvement, n (%)	31<br>(86.1)	230<br>(83.9)	ns
Cortical bone tracer uptake, n (%)	23<br>(88.5)	162<br>(94.6)	ns
Lung involvement, n (%)	15<br>(41.7)	57<br>(20.8)	**0.007**
Kidney/retroperitoneum involvement, n (%)	25<br>(69.4)	151<br>(55.1)	ns
Skin involvement, n (%)	9<br>(25.0)	83<br>(30.3)	ns
Serosal involvement, n (%)	19<br>(52.8)	75<br>(27.3)	**0.002**
Pleural involvement, n (%)	9<br>(25)	26<br>(9.5)	**0.007**
Pericardial involvement, n (%)	11<br>(30.6)	45<br>(17.2)	ns
Peritoneal involvement, n (%)	9<br>(25)	23<br>(8.4)	**0.002**
Reticuloendothelial system involvement, n (%)	8<br>(22.2)	13<br>(4.7)	**<0.001**

*Indicates those categories for which there is a statistically significant difference
Statistically significant p values are in bold

Table 5. Features of patients with mixed histiocytosis.

	**Patients with mixed histiocytosis**<br>**n= 26**	**Patients without mixed histiocytosis**<br>**n = 284**	**P value**
Age, mean ± SD	50.9<br>± 20.4	53.4<br>± 17.4	ns
Cardiovascular involvement, n (%)	11<br>(42.3)	137<br>(48.2)	ns
Central nervous system involvement, n (%)	12<br>(42.6)	183<br>(64.4)	ns
Bone involvement,<br>n (%)	23<br>(88.5)	238<br>(83.8)	ns
Cortical bone tracer uptake, n (%)	15<br>(93.8)	170<br>(93.8)	ns
Lung involvement,<br>n (%)	6<br>(23.1)	66<br>(23.2)	ns
Kidney/retroperitoneum involvement, n (%)	13<br>(50.0)	163<br>(57.4)	ns
Skin involvement,<br>n (%)	14<br>(53.8)	78<br>(27.5)	**0.005**
Papulonodular lesions, n (%)	9<br>(37.5)	21<br>(7.7)	**<0.001**
Serosal involvement,<br>n (%)	6<br>(23.1)	88<br>(30.1)	Ns
Pleural involvement,<br>n (%)	1<br>(3.8)	34<br>(12)	Ns
Pericardial involvement,<br>n (%)	2<br>(8.0)	54<br>(19.8)	Ns
Peritoneal involvement,<br>n (%)	3<br>(11.5)	29<br>(10.2)	Ns
Reticuloendothelial system involvement, n (%)	4<br>(15.4)	17<br>(6)	Ns

Statistically significant p values are in bold

META-ANALYSIS REFERENCES:

1. Blombery P, Wong SQ, Lade S, et al. Erdheim-Chester disease harboring the BRAF V600E mutation. J Clin Oncol. 2012 Nov 10;30(32):e331-2.
2. Szturz P, Hlavatý L, Prášek J, et al. Erdheim-Chester disease and Schnitzler syndrome: so near, and yet so far. Int J Hematol. 2013 Oct;98(4):379-81.
3. Kornik RI, Naik HB, Lee CC, et al. Diabetes insipidus, bone lesions, and new-onset red-brown papules in a 42-year-old man. J Am Acad Dermatol. 2013 Jun;68(6):1034-8.
4. Rouco I, Arostegui J, Cánovas A, et al. Neurological manifestations in Erdheim-Chester disease: Two case reports. Neurologia. 2016 Jul-Aug;31(6):426-8. English, Spanish.
5. Toro Galván S, Planas Vilaseca A, Michalopoulou Alevras T, et al. Endocrine changes in histiocytosis of the hypothalamic-pituitary axis. Endocrinol Nutr. 2015 Feb;62(2):72-9. English, Spanish.
6. Janku F, Vibat CR, Kosco K, et al. BRAF V600E mutations in urine and plasma cell-free DNA from patients with Erdheim-Chester disease. Oncotarget. 2014 Jun 15;5(11):3607-10.
7. Guo S, Yan Q, Rohr J, et al. Erdheim-Chester disease involving the breast--a rare but important differential diagnosis. Hum Pathol. 2015 Jan;46(1):159-64.
8. Bosco J, Allende A, Varikatt Wet al. Does the BRAF(V600E) mutation herald a new treatment era for Erdheim-Chester disease? A case-based review of a rare and difficult to diagnose disorder. Intern Med J. 2015 Mar;45(3):348-51.
9. Cangi MG, Biavasco R, Cavalli G, et al. BRAFV600E-mutation is invariably present and associated to oncogene-induced senescence in Erdheim-Chester disease. Ann Rheum Dis. 2015 Aug;74(8):1596-602.
10. Mazor RD, Manevich-Mazor M, Kesler A, et al. Clinical considerations and key issues in the management of patients with Erdheim-Chester Disease: a seven case series. BMC Med. 2014 Dec 1;12:221.

11. Haroche J, Cohen-Aubart F, Emile JF, et al. Reproducible and sustained efficacy of targeted therapy with vemurafenib in patients with BRAF(V600E) -mutated Erdheim-Chester disease. J Clin Oncol. 2015 Feb 10;33(5):411-8.
12. Emile JF, Diamond EL, Hélias-Rodzewicz Z, et al. Recurrent RAS and PIK3CA mutations in Erdheim-Chester disease. Blood. 2014 Nov 6;124(19):3016-9.
13. García-Gómez FJ, Cambil-Molina T, Ríos-Martín JJ, et al. Bone scintigraphy as cornerstone in the diagnosis of Erdheim-Chester disease. Rev Esp Med Nucl Imagen Mol. 2016 May-Jun;35(3):193-6. English, Spanish.
14. Bulycheva EN, Baykov VV, Zaraĭskiĭ MI, et al. Rare form of Erdheim-Chester disease presenting with isolated central skeletal lesions treated with a combination of Alfa-Interferon and zoledronic Acid. Case Rep Hematol. 2015;2015:876752
15. Taguchi S, Kishida Y, Tamura K, et al. Intrapelvic Bulky Tumor as an Unusual Presentation of Erdheim-Chester Disease. Intern Med. 2015;54(24):3241-5.
16. Elkouzi A, Rauschkolb P, Grogg KL, et al. Neurohistiocytosis of the Cerebellum: A Rare Cause of Ataxia. Mov Disord Clin Pract. 2015 Dec 11;3(2):125-129
17. Diamond EL, Durham BH, Haroche J, et al. Diverse and Targetable Kinase Alterations Drive Histiocytic Neoplasms. Cancer Discov. 2016 Feb;6(2):154-65.
18. Kim S, Lee M, Shin HJet al. Coexistence of intracranial Langerhans cell histiocytosis and Erdheim-Chester disease in a pediatric patient: a case report. Childs Nerv Syst. 2016 May;32(5):893-6.
19. Loh WJ, Sittampalam K, Tan SC, et al. Symptomatic empty sella syndrome: an unusual manifestation of Erdheim-Chester disease. Endocrinol Diabetes Metab Case Rep. 2015;2015:140122.
20. Ponsiglione A, Puglia M, Barbuto L, et al. Cardiac involvement in Erdheim- Chester disease: MRI findings and literature revision. Acta Radiol Open. 2015 Sep 7;4(9):2058460115592273.

21. Cives M, Simone V, Rizzo FM, et al. Erdheim-Chester disease: a systematic review. Crit Rev Oncol Hematol. 2015 Jul;95(1):1-11.
22. Euskirchen P, Haroche J, Emile JF. Complete remission of critical neurohistiocytosis by vemurafenib. Neurol Neuroimmunol Neuroinflamm. 2015 Feb 26;2(2):e78.
23. Schirmer JH, Thorns C, Moosig F, et al. Treatment failure by canakinumab in a patient with progressive multisystemic Erdheim-Chester disease refractory to anakinra: successful use of vemurafenib. Rheumatology (Oxford). 2015 Oct;54(10):1932-4.
24. Tzoulis C, Schwarzlmüller T, Gjerde IO, et al. Excellent response of intramedullary Erdheim-Chester disease to vemurafenib: a case report. BMC Res Notes. 2015 Apr 30;8:171.
25. Okamura K, Suematsu Y, Morizumi S, et al. Erdheim-Chester Disease With Cardiovascular Involvement and BRAF V600E Mutation. Circ J. 2016 Jun 24;80(7):1657-9.
26. Kyriakopoulou M, Decaux G, El Mourad M, et al. Acute Cardiac Tamponade in a 77-year-old Italian Woman with Erdheim-Chester Disease. Eur J Case Rep Intern Med. 2016 Sep 19;3(7):000451.
27. Borys D, Nystrom L, Song A, et al. Erdheim Chester disease with appendicular skeletal, renal and pleural involvement responding to Zelboraf (BRAF inhibitor) treatment: case report. Skeletal Radiol. 2016 Oct;45(10):1397-402.
28. Houston BA, Miller PE, Rooper LM, et al. CLINICAL PROBLEM-SOLVING. From Dancing to Debilitated. N Engl J Med. 2016 Feb 4;374(5):470-7.
29. Stempel JM, Bustamante Alvarez JG, Carpio AM, et al. Erdheim-Chester disease, moving away from the orphan diseases: A case report. Respir Med Case Rep. 2016 Dec 3;20:55-58
30. Al Bayati A, Plate T, Al Bayati M, et al. Dabrafenib and Trametinib Treatment for Erdheim-Chester Disease With Brain Stem Involvement. Mayo Clin Proc Innov Qual Outcomes. 2018 Jul 4;2(3):303-308.

31. Liao X, Thorson JA, Hughes T, et al. Erdheim-Chester disease with novel gene mutations discovered as an incidental finding in explanted liver of a patient with hepatitis C cirrhosis: A case report and literature review. Pathol Res Pract. 2016 Sep;212(9):849-54.
32. Dave AA, Gutschow SE, Walker CM. A Case of Incidentally-diagnosed Erdheim-Chester Disease. Cureus. 2016 Sep 13;8(9):e781.
33. Bradshaw MJ, Pawate S, Bloch KC, et al. Clinical Reasoning: A 52-year-old man with diplopia and ataxia. Neurology. 2016 Sep 27;87(13):e140-3.
34. Neckman JP, Kim J, Mathur M, et al. Diverse cutaneous manifestations of Erdheim-Chester disease in a woman with a history of Langerhans cell histiocytosis. JAAD Case Rep. 2016 Mar 5;2(2):128-31.
35. Diamond EL, Abdel-Wahab O, Durham BH, et al. Anakinra as efficacious therapy for 2 cases of intracranial Erdheim-Chester disease. Blood. 2016 Oct 6;128(14):1896-1898.
36. Cao XX, Sun J, Li J, et al. Evaluation of clinicopathologic characteristics and the BRAF V600E mutation in Erdheim-Chester disease among Chinese adults. Ann Hematol. 2016 Apr;95(5):745-50.
37. Zhu P, Li N, Yu L, et al. Erdheim-Chester Disease with Emperipolesis: A Unique Case Involving the Heart. Cancer Res Treat. 2017 Apr;49(2):553-558.
38. Nordmann TM, Juengling FD, Recher M, et al. Trametinib after disease reactivation under dabrafenib in Erdheim-Chester disease with both *BRAF* and *KRAS* mutations. Blood. 2017 Feb 16;129(7):879-882.
39. Hunt D, Milne P, Fernandes P, et al. Targeted treatment of brainstem neurohistiocytosis guided by urinary cell-free DNA. Neurol Neuroimmunol Neuroinflamm. 2016 Nov 3;4(1):e299.
40. Gianfreda D, Palumbo AA, Rossi E, et al. Cardiac involvement in Erdheim-Chester disease: an MRI study. Blood. 2016 Nov 17;128(20):2468-2471.

41. Gianfreda D, Musetti C, Nicastro M, et al. Erdheim-Chester Disease as a Mimic of IgG4-Related Disease: A Case Report and a Review of a Single-Center Cohort. Medicine (Baltimore). 2016 May;95(21):e3625.
42. Iurlo A, Dagna L, Cattaneo Det al. Erdheim-Chester Disease With Multiorgan Involvement, Following Polycythemia Vera: A Case Report. Medicine (Baltimore). 2016 May;95(20):e3697.
43. Windisch C, Petersen I, Schulz B, et al. Erdheim-Chester disease with vascular involvement mimics large vessel vasculitis. Postgrad Med J. 2016 Nov;92(1093):687-688.
44. Nadjiri J, Woertler K, Specht K, et al. Erdheim-Chester disease with bilateral Achilles tendon involvement. Skeletal Radiol. 2016 Oct;45(10):1437-42.
45. Cohen Aubart F, Emile JF, Maksud P, et al. Efficacy of the MEK inhibitor cobimetinib for wild-type BRAF Erdheim-Chester disease. Br J Haematol. 2018 Jan;180(1):150-153.
46. Cohen-Aubart F, Maksud P, Saadoun D, et al. Variability in the efficacy of the IL1 receptor antagonist anakinra for treating Erdheim-Chester disease. Blood. 2016 Mar 17;127(11):1509-12
47. Franconieri F, Martin-Silva N, de Boysson H, et al. Superior efficacy and tolerance of reduced doses of vemurafenib plus anakinra in Erdheim-Chester disease: Towards the paradigm of combined targeting and immune therapies. Acta Oncol. 2016 Jul;55(7):930-2.
48. Sagnier S, Debruxelles S, Lepreux S, et al. Erdheim-Chester Disease: An Unusual Cause of Intracranial Vasculitis and Progressive Leukoencephalopathy. J Stroke Cerebrovasc Dis. 2016 May;25(5):e63-e65.
49. Jean-Michel V, Coustans M, Wehbe B, et al. Erdheim Chester disease: A rare obstructive case of acute renal failure. Presse Med. 2016 Nov;45(11):1066-1069.
50. Parreau S, Haroche J, Pommepuy I, et al. Langerhans cell histiocytosis and Erdheim-Chester disease, a continuity? Rev Med Interne. 2017 Jul;38(7):482-487. French.

51. Benoist N, Mikail N, Deschamps L, et al. Erdheim-Chester disease as assessed by modern multimodality imaging. Int J Cardiol. 2016 Mar 15;207:235-7.
52. Binyousef RF, Al-Gahmi AM, Khan ZR, et al. A rare case of Erdheim-Chester disease in the breast. Ann Saudi Med. 2017 Jan-Feb;37(1):79-83.
53. Salama H, Kojan S, Abdulrahman S, et al Erdheim-Chester Disease with No Skeletal Bone Involvement and Massive Weight Loss. Case Rep Hematol. 2017; 2017:3862052
54. Khan MR, Ashraf MS, Belgaumi AF. Erdheim Chester disease: An unusual presentation of a rare histiocytic disease in a 3-year old boy. Pediatric Hematology Oncology Journal 2 (2017) 59e62
55. Pacios Blanco RE, Gorospe Sarasua L, Reguero ME. Spontaneous Bilateral Pneumothoraces in Erdheim-Chester Disease. Arch Bronconeumol (Engl Ed). 2018 Apr;54(4):219. English, Spanish.
56. Ortega Zufiría JM, Choque Cuba B, Poveda Núñez P, et al. Erdheim-Chester disease with meningeal involvement: A case report. Neurocirugia (Astur). 2017 May-Jun;28(3):157-158. Spanish.
57. Váradi Z, Bánusz R, Csomor J, et al. Effective BRAF inhibitor vemurafenib therapy in a 2-year-old patient with sequentially diagnosed Langerhans cell histiocytosis and Erdheim-Chester disease. Onco Targets Ther. 2017 Jan 24;10:521-526.
58. Chasseur P, Kyriakopoulou M, Vokaer B, et al. A cytologic diagnosis of $BRAF^{V600E}$ Erdheim-Chester disease on pericardial fluid. Acta Clin Belg. 2017 Oct;72(5):369-371. Ù
59. Goyal G, Shah MV, Call TG, et al. Clinical and radiological responses to oral methotrexate alone or in combination with other agents in Erdheim-Chester disease. Blood Cancer J. 2017 Dec 15;7(12):647.
60. Goyal G, Shah MV, Call TG, et al. Clinical and Radiologic Responses to Cladribine for the Treatment of Erdheim-Chester Disease. JAMA Oncol. 2017 Sep 1;3(9):1253-1256.

61. Pan Z, Kleinschmidt-DeMasters BK. CNS Erdheim-Chester Disease: A Challenge to Diagnose. J Neuropathol Exp Neurol. 2017 Dec 1;76(12):986-996.
62. Balasubramanian G, Modiri A, Affi M, et al. A Fatal Case of Erdheim-Chester Disease with Hepatic Involvement. ACG Case Rep J. 2017 Aug 2;4:e95.
63. Gupta A, Yeganeh A, Rootman D, et al. Vemurafenib (BRAF Inhibitor) Therapy for Orbital Erdheim-Chester Disease. Ophthalmic Plast Reconstr Surg. 2017 Nov/Dec;33(6):e138-e139.
64. Tan ACS, Yzer S, Atebara N, et al. Three Cases of Erdheim-Chester Disease With Intraocular Manifestations: Imaging and Histopathology Findings of a Rare Entity. Am J Ophthalmol. 2017 Apr;176:141-147.
65. Nikonova A, Esfahani K, Chausse G, et al. Erdheim-Chester Disease: The Importance of Information Integration. Case Rep Oncol. 2017 Jul 11;10(2):613-619.
66. Chen M, Ding C, Lu T, et al. Langerhans cell histiocytosis and Erdheim-Chester disease overlap syndrome with bone marrow involvement and type 2 diabetes mellitus. Ann Hematol. 2018 Jan;97(1):189-192.
67. Techavichit P, Sosothikul D, Chaichana T, et al. BRAF V600E mutation in pediatric intracranial and cranial juvenile xanthogranuloma. Hum Pathol. 2017 Nov;69:118-122.
68. Milne P, Bigley V, Bacon CM, et al. Hematopoietic origin of Langerhans cell histiocytosis and Erdheim-Chester disease in adults. Blood. 2017 Jul 13;130(2):167-175.
69. Chiapparini L, Cavalli G, Langella T, et al. Adult leukoencephalopathies with prominent infratentorial involvement can be caused by Erdheim-Chester disease. J Neurol. 2018 Feb;265(2):273-284.
70. Liersch J, Carlson JA, Schaller J. Histopathological and Clinical Findings in Cutaneous Manifestation of Erdheim-Chester Disease and Langerhans Cell Histiocytosis Overlap Syndrome Associated With the BRAFV600E Mutation. Am J Dermatopathol. 2017 Jul;39(7):493-503.

71. Papo M, Diamond EL, Cohen-Aubart F, et al. High prevalence of myeloid neoplasms in adults with non-Langerhans cell histiocytosis. Blood. 2017 Aug 24;130(8):1007-1013.
72. Razanamahery J, Jacquier A, Humbert S. A Rare Case of Chylous Ascites. Gastroenterology. 2017 Oct;153(4):903-905.
73. Fargeot G, Stefanizzi S, Depuydt S, et al. Association between Ischemic Stroke and Erdheim-Chester Disease: A Case Report and Review of Literature. J Stroke Cerebrovasc Dis. 2017 Aug;26(8):e153-e155.
74. Quinaglia-Silva T, Medina F, Ramos CD, et al. Cardiac Involvement in Erdheim-Chester Disease. Circ Cardiovasc Imaging. 2018 Dec;11(12):e008531.
75. Costa IBSDS, Abdo ANR, Bittar CS, et al. Cardiovascular Manifestations of Erdheim-Chester's Disease: A Case Series. Arq Bras Cardiol. 2018 Dec;111(6):852-855.
76. Toya T, Ogura M, Toyama K, et al. Prognostic factors of Erdheim-Chester disease: a nationwide survey in Japan. Haematologica. 2018 Nov;103(11):1815-1824.
77. Tamura S, Kawamoto K, Miyoshi H, et al. Cladribine treatment for Erdheim-Chester disease involving the central nervous system and concomitant polycythemia vera: A case report. J Clin Exp Hematop. 2018 Dec 13;58(4):161-165.
78. Verschelden G, Van Laethem J, Velkeniers B, et al. Significant response to dabrafenib in a patient with Erdheim-Chester disease with BRAFV600E mutation. Pol Arch Intern Med. 2018 Jun 29;128(6):386-388.
79. Jouni H, Kuzo RS, Anavekar NS. Solving a Mystery . . . 8 Years Later. J Investig Med High Impact Case Rep. 2018 Jan 24;6:2324709617752962.
80. Picarsic J, Pysher T, Zhou H, et al. BRAF V600E mutation in Juvenile Xanthogranuloma family neoplasms of the central nervous system (CNS-JXG): a revised diagnostic algorithm to include pediatric Erdheim-Chester disease. Acta Neuropathol Commun. 2019 Nov 4;7(1):168.

81. Sakr HI, Buckley K, Baiocchi R, et al. Erdheim Chester disease in a patient with Burkitt lymphoma: a case report and review of literature. Diagn Pathol. 2018 Nov 24;13(1):94.
82. Huang LC, Topping KL, Gratzinger D, et al. Orbital and chorioretinal manifestations of Erdheim-Chester disease treated with vemurafenib. Am J Ophthalmol Case Rep. 2018 Jul 25;11:158-163.
83. Ozkaya N, Rosenblum MK, Durham BH, et al. The histopathology of Erdheim-Chester disease: a comprehensive review of a molecularly characterized cohort. Mod Pathol. 2018 Apr;31(4):581-597.
84. Hao X, Feng R, Bi Y, et al. Dramatic efficacy of dabrafenib in Erdheim-Chester disease (ECD): a pediatric patient with multiple large intracranial ECD lesions hidden by refractory Langerhans cell histiocytosis. J Neurosurg Pediatr. 2018 Sep 28;23(1):48-53.
85. Tzankov A, Kremer M, Leguit R, et al. Histiocytic cell neoplasms involving the bone marrow: summary of the workshop cases submitted to the 18th Meeting of the European Association for Haematopathology (EAHP) organized by the European Bone Marrow Working Group, Basel 2016. Ann Hematol. 2018 Nov;97(11):2117-2128.
86. Zanelli M, Smith M, Mengoli MC, Spaggiari L, De Marco L, Lococo F, Puma F, Ascani S. Erdheim-Chester disease: description of two illustrative cases involving the lung. Histopathology. 2018 Jul;73(1):167-172.
87. Tomelleri A, Cavalli G, De Luca G, et al. Treating Heart Inflammation With Interleukin-1 Blockade in a Case of Erdheim-Chester Disease. Front Immunol. 2018 Jun 1;9:1233.
88. Özden F, Schinke S, Thorns C, et al. Detection of anti-neutrophil cytoplasmic and antinuclear autoantibodies favouring misdiagnoses in 5 cases of Erdheim-Chester disease. Clin Exp Rheumatol. 2018 Mar-Apr;36 Suppl 111(2):176. Epub 2018 Apr 10.
89. Knitza J, Kampylafka E, Wacker J, et al. Morbus Erdheim-Chester: Eine wichtige Differenzialdiagnose und ihre Leitsymptome [Erdheim-Chester disease: An important differential diagnosis and its main symptoms]. Z Rheumatol. 2019 Feb;78(1):66-71.

90. Franconieri F, Deshayes S, de Boysson H, et al. Superior efficacy and similar safety of double dose anakinra in Erdheim-Chester disease after single dose treatment. Oncoimmunology. 2018 Apr 9;7(8):e1450712.
91. Bunaux K, Sevestre H, Emile JF, et al. A case of Erdheim-Chester disease with spinal cord compression and sphenoid sinus involvement. Neurochirurgie. 2018 Dec;64(6):439-441.
92. Fernández-Eulate G, Muñoz-Lopetegi A, Ruiz I, et al. Vemurafenib as first-line therapy in BRAF-V600E-mutant Erdheim-Chester disease with CNS involvement. BMJ Case Rep. 2019 Nov 19;12(11):e228280.
93. Sánchez-Villalobos JM, Jimeno-Almazán A, López-Peña C, et al. Erdheim-Chester disease mimicking multiple sclerosis or a new association? Mult Scler Relat Disord. 2019 May;30:94-97.
94. Pivkova-Veljanovska A, Ivanovski M, Panovska-Stavridis I, et al. A Rare Case of Soft Tissue Erdheim Chester Disease: Diagnostic Dilemma and Management. Open Access Maced J Med Sci. 2019 Jun 5;7(11):1808-1811.
95. Ghobadi A, Miller CA, Li T, et al. Shared cell of origin in a patient with Erdheim-Chester disease and acute myeloid leukemia. Haematologica. 2019 Aug;104(8):e373-e375.
96. Liew JW, Starkebaum G. Bone Lesions in Erdheim-Chester Disease. Arthritis Rheumatol. 2019 Jul;71(7):1206.
97. Braue JA, Al-Rohil RN. Yellow Periorbital Plaque and Retroperitoneal Fibrosis. JAMA Dermatol. 2019 Apr 1;155(4):483-484.
98. Ding F, Chahine J, Deshwal H, et al. Mysterious quad of constrictive pericarditis, recurrent pleural effusions, bone involvement and interstitial lung disease. Oxf Med Case Reports. 2019 Mar 29;2019(3):omz015.
99. Durham BH, Lopez Rodrigo E, Picarsic J, et ak. Activating mutations in CSF1R and additional receptor tyrosine kinases in histiocytic neoplasms. Nat Med. 2019 Dec;25(12):1839-1842.

100. Wang F, Cao X, Niu N, et al. Multisystemic Imaging Findings in Chinese Patients with Erdheim-Chester Disease. AJR Am J Roentgenol. 2019 Dec;213(6):1179-1186.

101. Jang JH, Oh J, Shim HS, Kang SM. Unusual Case of Recurrent Pericardial Effusion - Erdheim-Chester Disease. Circ J. 2019 Jul 25;83(8):1762.

102. Wang JN, Qiu Y, Niu N, et al. Successful treatment of central nervous system involved Erdheim-Chester disease by intermediate-dose cytarabine as first-line therapy. Acta Oncol. 2020 Mar;59(3):302-305.

103. Buono A, Bassi I, Santolamazza C, et al. Getting to the heart of the matter in a multisystem disorder: Erdheim-Chester disease. Lancet. 2019 Aug 17;394(10198):e19.

104. Zanelli M, Smith M, Mengoli MC, et al. Erdheim-Chester disease: description of two illustrative cases involving the lung. Histopathology. 2018 Jul;73(1):167-172.

105. Todisco A, Cavaliere C, Vaglio A, et al. Erdheim-Chester disease: A challenging diagnosis for an effective therapy. Clin Neurol Neurosurg. 2020 Jul;194:105841.

106. Miron G, Karni A, Faust-Soher A, et al. Erdheim-Chester disease presenting with chorea and mimicking IgG4-related disorder. Neurol Clin Pract. 2019 Dec;9(6):524-526.

107. Bonnet P, Chasset F, Moguelet P, et al. Erdheim-Chester disease associated with chronic myelomonocytic leukemia harboring the same clonal mutation. Haematologica. 2019 Nov;104(11):e530-e533.

108. Cadour F, De Masi-Jacquier M, Barral PA, et al. Thoracic Aortic Aneurysms and Erdheim-Chester Disease. J Vasc Interv Radiol. 2019 Oct;30(10):1698-1700.

109. Kai K, Komohara Y, Shinojima N, et al. A case of suprasellar Erdheim-Chester disease and characterization of macrophage phenotype. J Clin Exp Hematop. 2020 Dec 15;60(4):179-182.

110. Kemps PG, Hebeda KM, Pals ST, et al. Spectrum of histiocytic neoplasms associated with diverse haematological malignancies bearing the same oncogenic mutation. J Pathol Clin Res. 2021 Jan;7(1):10-26.

111. Papageorgiou SG, Divane A, Roumelioti M, et al. Erdheim-Chester Disease and Acute Myeloid Leukemia with Mutated NPM1 in a Patient with Clonal Hematopoiesis: A Case Report. Onco Targets Ther. 2020 Nov 16;13:11689-11695.

112. Goyal G, Ravindran A, Liu Y, et al. Mayo Clinic Histiocytosis Working Group. Bone marrow findings in Erdheim-Chester disease: increased prevalence of chronic myeloid neoplasms. Haematologica. 2020 Jan 31;105(2):e84-e86.

113. Budhram A, Rech KL, Peikert JM, et al. Teaching NeuroImages: Brain and Skin Involvement in Erdheim-Chester Disease. Neurology. 2021 Mar 16;96(11):e1590-e1592.

114. Simpson HD, Aksamit AJ, Zalewski NL. Longitudinally Extensive Spinal Cord Lesion in Erdheim-Chester Disease. JAMA Neurol. 2020 Nov 1;77(11):1446-1447.

115. Ruan GJ, Goyal G, Go RS. 43-Year-Old Man With Polyuria and Bone Pain. Mayo Clin Proc. 2020 Feb;95(2):e13-e18.

116. Sanchez-Nadales A, Anampa-Guzman A, Navarro-Motta J. Erdheim-Chester Disease With Extensive Pericardial Involvement: A Case Report and Systematic Review. Cardiol Res. 2020 Apr;11(2):118-128.

117. Yoo J, Gunsteen C, Patel S, et al. Kidney Transplantation for Erdheim-Chester Disease. Case Rep Transplant. 2020 Jul 13;2020:3954165.

118. Gray JCR, Kim J, Digianvittorio M, et al. BRAF-Mutated Erdheim-Chester Disease: Profound Response to Vemurafenib Visualized With Serial Multimodality Imaging. J Natl Compr Canc Netw. 2020 Jun;18(6):650-655.

119. De la Fuente MI, Rosenblum MK, Diamond EL, et al. Erdheim-Chester disease among neuroinflammatory syndromes: the case for precision medicine. Neurol Neuroimmunol Neuroinflamm. 2020 Mar 2;7(3):e686.

120. He T, Cui L, Niu N, et al. Bone mineral density and bone microarchitecture in a cohort of patients with Erdheim-Chester Disease. Orphanet J Rare Dis. 2020 Sep 4;15(1):236.

121. Yang Z, Zhao S, Zhou J, et al. Erdheim-Chester disease: a case treated with IFN-α monitored using plasma and urine cell-free DNA. Immunotherapy. 2020 Apr;12(6):379-387.

122. Brodie J, Zhou S, Makkuni D, et al. Erdheim-Chester Disease: Two cases from an ophthalmic perspective. Am J Ophthalmol Case Rep. 2020 Nov 2;20:100984.

123. Pegoraro F, Maniscalco V, Peyronel F, et al. Long-term follow-up of mTOR inhibition for Erdheim-Chester disease. Blood. 2020 May 28;135(22):1994-1997.

124. Klain M, Schlumberger M, Cuocolo A. Concurrent BRAF V600E mutated papillary thyroid carcinoma and Erdheim-Chester disease. Endocrine. 2020 Dec;70(3):655-656.

125. Cerudelli E, Gazzilli M, Bertoli M, et al. Erdheim-Chester disease: The power of nuclear medicine imaging. Rev Esp Med Nucl Imagen Mol (Engl Ed). 2020 Sep-Oct;39(5):323-324. English, Spanish.

126. Marano M, Todisco A, Motolese F, et al. Choreo-Athetosis and Ataxia as Leading Features in a Case of Erdheim-Chester Disease. Mov Disord Clin Pract. 2020 Jan 22;7(2):215-217.

127. Mazor RD, Weissman R, Luckman J, et al. Dual BRAF/MEK blockade restores CNS responses in BRAF-mutant Erdheim-Chester disease patients following BRAF inhibitor monotherapy. Neurooncol Adv. 2020 Mar 3;2(1):vdaa024.

128. Papo M, Corneau A, Cohen-Aubart F, et al. Immune phenotyping of Erdheim-Chester disease through mass cytometry highlights decreased proportion of non-classical monocytes and increased proportion of Th17 cells. Ann Rheum Dis. 2020 Nov;79(11):1522-1524.

129. Berthe P, Rouzic N, Daelman L, et al. Utilisation de thérapie ciblée dans la maladie d'Erdheim-Chester : à propos d'un cas avec atteinte du système nerveux central [Use of targeting therapy in Erdheim-Chester disease: A case report with neurologic involvement]. Rev Med Interne. 2020 Jun;41(6):413-417.

130. Wu S, Pang Y, Chen Y, et al. 68Ga-DOTA-FAPI-04 PET/CT in Erdheim-Chester Disease. Clin Nucl Med. 2021 Mar 1;46(3):258-260.

131. Lauricella E, d'Amati A, Ingravallo G, et al. Cerebellar ataxia and exercise intolerance in Erdheim-Chester disease. Cerebellum Ataxias. 2021 Jan 6;8(1):3.

132. Cui R, Chen M, Dai SM. Coated aorta in Erdheim-Chester disease. Rheumatology (Oxford). 2021 Feb 1;60(2):986-987.

133. Ammad Ud Din M, Hussain SA, Phatak PD. Bilateral Perinephric Stranding and Diffuse Bone Lesions. Am J Med Sci. 2021 Jul;362(1):e5-e6.
