# Supplementary material for: Genetics and clinical phenotype of Erdheim–Chester disease: A case report of constrictive pericarditis and a systematic review of the literature
Source: Front Cardiovasc Med. 2022 Aug 11;9:876294. doi: 10.3389/fcvm.2022.876294 (PMC9403274; doi:10.3389/fcvm.2022.876294)
Supplement: Supplementary file 2 [file Data_Sheet_2.pdf]

Supplementary box

***Box 1*, Red flags of Erdheim-Chester disease.**

- Bone involvement (typically lower-extremity bone pain with nuclear imaging showing bilateral symmetric osteosclerosis).
- Cardiovascular involvement (right atrioventricular infiltration (“pseudotumor”), periaortic infiltration (“coated aorta”), pericardial infiltration with effusion and, less frequently, constrictive pericarditis).
- Central nervous system involvement (dura and parenchymal involvement).
- Retroorbital infiltration.
- Pituitary involvement.
- Reticuloendothelial system involvement (liver, spleen, lymph nodes, bone marrow).
- Associated hematological disease (typically, chronic myeloproliferative neoplasm, but also acute, lymphoid, dysplastic or another histiocytic disorder).
- Skin lesions (typically xanthelasma, less frequently papulonodular lesions).
- Retroperitoneal and peritoneal involvement (peri-kidney infiltration (“hairy kidney”), adrenal glands and peritoneum infiltration).
- Lung involvement (parenchyma and pleura).
