# Supplementary material for: Genetics and clinical phenotype of Erdheim–Chester disease: A case report of constrictive pericarditis and a systematic review of the literature
Source: Front Cardiovasc Med. 2022 Aug 11;9:876294. doi: 10.3389/fcvm.2022.876294 (PMC9403274; doi:10.3389/fcvm.2022.876294)
Supplement: Supplementary file 4 [file Table_2.pdf]

Supplementary Table 2: Quality assessment of the studies:

No.	Author	Year	Selection	Ascertainment	Causality	Reporting		Overall judgment
1	Blombery et al	2012	Good	Good	Good	Good		Good
2	Kornik et al	2013	Good	Good	Good	Good		Good
3	Szturz et al	2013	Good	Good	Good	Good		Good
4	Rouco et al	2014	Moderate	Good	Good	Good		Good
5	Galvan et al	2014	Low	Moderate	Good	Good		Moderate
6	Janku et al	2014	Good	Low	Good	Moderate		Moderate
7	Guo e t al	2014	Good	Good	Good	Good		Good
8	Bosco et al	2014	Good	Good	Good	Good		Good
9	Furuta et al	2014	Good	Good	Good	Good		Good
10	Cangi et al	2014	Moderate	Low	Good	Moderate		Moderate
11	Mazor et al	2014	Moderate	Moderate	Good	Good		Moderate
12	Haroche et al	2014	Moderate	Low	Good	Good		Moderate
13	Emile et al	2014	Moderate	Good	Good	Low		Moderate
14	Garcia Gomez et al	2015	Good	Good	Good	Good		Good

15	Bulycheva et al	2015	Good	Good	Good	Good		Good
16	Taguchi et al	2015	Good	Moderate	Good	Good		Good
17	Elkouzi et al	2015	Good	Good	Good	Good		Good
18	Diamond et al	2015	Low	Low	Good	Moderate		Moderate
19	Kim et al	2015	Good	Moderate	Good	Good		Good
20	Loh et al	2015	Good	Good	Good	Good		Good
21	Ponsiglione et al	2015	Good	Moderate	Good	Good		Good
22	Cives et al	2015	Good	Good	Good	Good		Good
23	Euskirchen et al	2015	Good	Good	Good	Good		Good
24	Schirmer et al	2015	Good	Moderate	Good	Good		Good
25	Tzoulis et al	2015	Good	Good	Good	Good		Good
26	Okamura et al	2016	Good	Good	Good	Good		Good
27	Borys et al	2016	Good	Good	Good	Good		Good
28	Houston et al	2016	Good	Good	Good	Good		Good
29	Stempel et al	2016	Good	Good	Good	Good		Good
30	Al Bayati et Al	2016	Good	Good	Good	Good		Good

31	Kyriakopoulou et al	2016	Good	Moderate	Good	Good		Good
32	Dave et al	2016	Good	Low	Good	Good		Moderate
33	Bradshaw et al	2016	Good	Moderate	Good	Good		Good
34	Neckman et al	2016	Moderate	Good	Good	Good		Good
35	Diamond et al	2016	Moderate	Good	Good	Good		Good
36	Zhu et al	2016	Good	Good	Good	Good		Good
37	Nordmann et al	2016	Good	Good	Good	Good		Good
38	Hunt et al	2016	Good	Good	Good	Good		Good
39	Iurlo et al	2016	Good	Good	Good	Good		Good
40	Windisch et al	2016	Good	Good	Good	Good		Good
41	Nadijiri et al	2016	Good	Good	Good	Good		Good
42	Franconieri et al	2016	Moderate	Good	Good	Moderate		Good
43	Sagnier et al	2016	Good	Good	Good	Good		Good
44	Jean-Michel et al	2016	Good	Good	Good	Good		Good
45	Parreau et al	2016	Good	Good	Good	Good		Good
46	Liao et al	2016	Good	Good	Good	Good		Good

47	Gianfreda et al	2016	Moderate	Moderate	Good	Moderate		Moderate
48	Cohen-Aubart et al	2016	Moderate	Low	Good	Moderate		Moderate
49	Benoits et al	2016	Good	Good	Good	Good		Good
50	Cao et all	2016	Moderate	Low	Good	Low		Moderate
51	Binyousef et al	2017	Good	Moderate	Good	Good		Good
52	Salama et al	2017	Good	Moderate	Good	Good		Good
53	Tan et al	2017	Moderate	Good	Good	Good		Good
54	Khan et al	2017	Good	Good	Good	Good		Good
55	Blanco et al	2017	Good	Moderate	Good	Good		Good
56	Zufiria et al	2017	Good	Moderate	Good	Good		Good
57	Varadi et al	2017	Good	Good	Good	Good		Good
58	Chasseur et al	2017	Good	Good	Good	Good		Good
59	Gupta et al	2017	Good	Moderate	Good	Good		Good
60	Nikonova et al	2017	Good	Good	Good	Good		Good
61	Chen et al	2017	Good	Good	Good	Good		Good
62	Razanamahery et al	2017	Good	Good	Good	Good		Good

63	Fargeot et al	2017	Good	Moderate	Good	Good		Good
64	Balasubramanian et al	2017	Good	Good	Good	Good		Good
65	Goyal et al	2017	Good	Good	Good	Good		Good
66	Goyal et al	2017	Moderate	Moderate	Good	Moderate		Moderate
67	Pan et al	2017	Good	Moderate	Good	Good		Good
68	Techavichit et al	2017	Good	Good	Good	Good		Good
69	Milne et al	2017	Good	Low	Good	Moderate		Moderate
70	Chiapparini et al	2017	Moderate	Moderate	Good	Moderate		Moderate
71	Liersch et al	2017	Good	Moderate	Good	Good		Good
72	Papo et al	2017	Moderate	Good	Good	Low		Moderate
73	Franconieri et al	2018	Moderate	Good	Good	Moderate		Good
74	Knitza et al	2018	Moderate	Moderate	Good	Good		Moderate
75	Oezden at al	2018	Low	Moderate	Good	Moderate		Moderate
76	Tomelleri et al	2018	Good	Good	Good	Good		Good
77	Tzankow et al	2018	Moderate	Moderate	Good	Good		Moderate
78	Hao et al	2018	Good	Good	Good	Good		Good

79	Ozkaya et al	2018	Moderate	Moderate	Good	Moderate		Moderate
80	Huang et al	2018	Good	Good	Good	Goog		Good
81	Sakr et al	2018	Good	Good	Good	Goog		Good
82	Picarsic et al	2018	Good	Moderate	Good	Good		Good
83	Jouni et al	2018	Good	Good	Good	Good		Good
84	Verschelden et al	2018	Good	Good	Good	Good		Good
85	Tamura et al	2018	Good	Good	Good	Good		Good
86	Bunaux et al	2018	Good	Good	Good	Good		Good
87	Toya et al	2018	Good	Low	Good	Good		Moderate
88	Costas et al	2018	Moderate	Good	Good	Good		Good
89	Silva et al	2018	Good	Good	Good	Good		Good
90	Cadour et al	2019	Good	Good	Good	Good		Good
91	Miron et al	2019	Moderate	Moderate	Good	Good		Moderate
92	Bonnet et al	2019	Moderate	Good	Good	Good		Good
93	Todisco et al	2019	Good	Good	Good	Good		Good
94	Durham et al	2019	Low	Good	Good	Good		Good

95	Zanelli et al	2019	Moderate	Moderate	Good	Good		Moderate
96	Liew et al	2019	Good	Moderate	Good	Good		
97	Buono et al	2019	Good	Moderate	Good	Good		Good
98	Wang et al	2019	Moderate	Moderate	Good	Good		Moderate
99	Jang et al	2019	Good	Moderate	Good	Good		Good
100	Wang et al	2019	Moderate	Low	Good	Moderate		Moderate
101	Ding et al	2019	Good	Moderate	Good	Good		Good
102	Braue, et al	2019	Good	Moderate	Good	Good		Good
103	Ghobadi et al	2019	Good	Good	Good	Good		Good
104	Pivkova-Veljanovska et al	2019	Good	Good	Good	Good		Good
105	van Bommel et al	2019	Good	Good	Good	Good		Good
106	Villalobos et al	2019	Good	Good	Good	Good		Good
107	Eulate et al	2019	Good	Moderate	Good	Good		Good
108	Berthe et al	2020	Good	Moderate	Good	Good		Good
109	Papo et al	2020	Moderate	Moderate	Moderate	Low		Moderate
110	Mazor et al	2020	Moderate	Good	Good	Good		Good

111	Kemps et al	2020	Good	Good	Good	Good		Good
112	Marano et al	2020	Good	Good	Good	Good		Good
113	Cerudelli et al	2020	Good	Good	Good	Good		Good
114	Klain et al	2020	Good	Good	Good	Good		Good
115	Pegoraro et al	2020	Moderate	Moderate	Good	Low		Moderate
116	Brodie et al	2020	Good	Good	Good	Good		Good
117	Yang et al	2020	Good	Good	Good	Good		Good
118	He et al	2020	Good	Moderate	Good	Good		Good
119	De la Fuente et al	2020	Good	Good	Good	Good		Good
120	Gray et al	2020	Good	Good	Good	Good		Good
121	Yoo et al	2020	Good	Moderate	Good	Good		Good
122	Sanchez-Nadales et al	2020	Good	Moderate	Good	Good		Good
123	Ruan et al	2020	Good	Good	Good	Good		Good
124	Simpson et al	2020	Good	Good	Good	Good		Good
125	Budhram et al	2020	Good	Good	Good	Good		Good
126	Goyal et al	2020	Moderate	Moderate	Good	Good		Moderate

127	Papageorgiou et al	2020	Good	Good	Good	Good		Good
128	Keitaro et al	2020	Good	Good	Good	Good		Good
129	Wu et al	2021	Good	Good	Good	Moderate		Good
131	Lauricella et al	2021	Good	Good	Good	Good		Good
132	Cui et al	2021	Good	Good	Good	Moderate		Good
133	Ud din et al	2021	Good	Good	Good	Moderate		Good
