# Supplementary figures and images for: Genetics and clinical phenotype of Erdheim–Chester disease: A case report of constrictive pericarditis and a systematic review of the literature
Source: Front Cardiovasc Med. 2022 Aug 11;9:876294. doi: 10.3389/fcvm.2022.876294 (PMC9403274; doi:10.3389/fcvm.2022.876294)

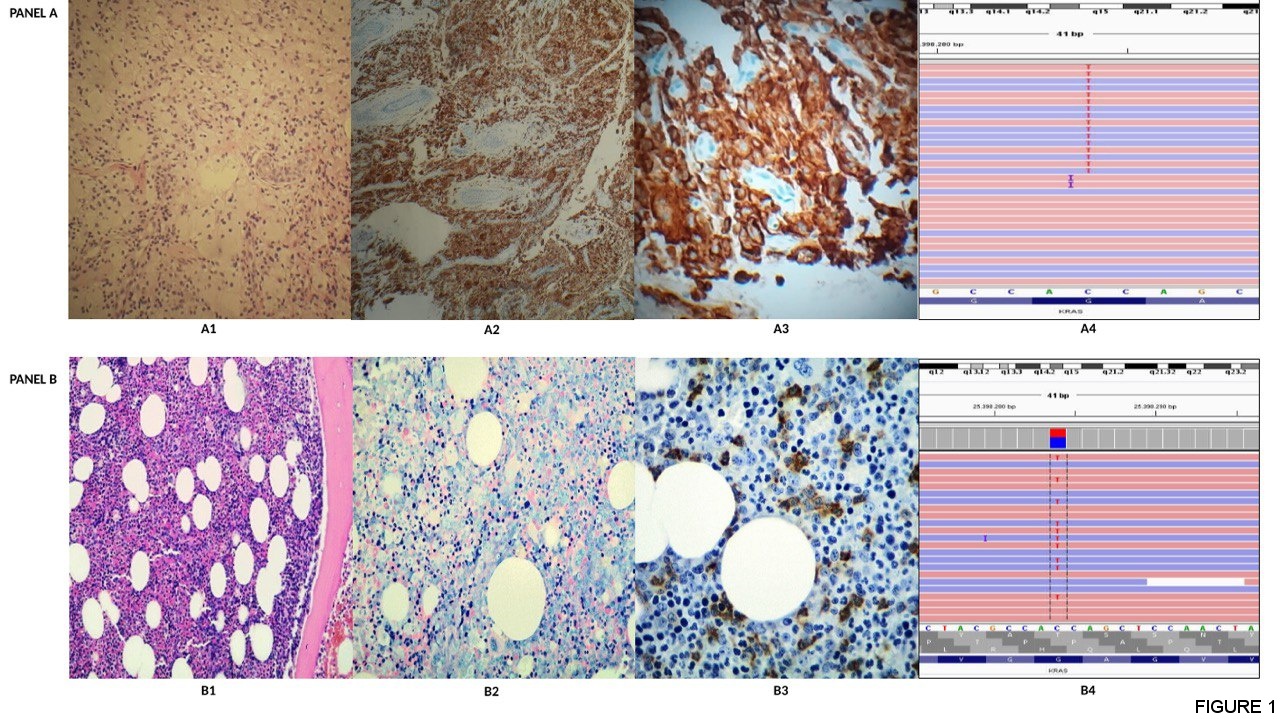

Supplement: Supplementary Figure 1 — (A) Biopsy of the eyelid. Images of hematoxylin-eosin stain (A1), CD 68 (A2), and CD163 (A3) immunohistochemistry. The illustrations show foamy, CD68+ and CD163+ histiocytes, typical of Erdheim-Chester disease. In the last image (A4), demonstration of a KRAS exon 2 mutation (p.Gly12Asp) by NGS (S5 Gene Studio) in the specimen. (B) Biopsy of the bone marrow (BM). Images of hematoxylin-eosin (B1), and May–Grünwald (B2) stain and CD14 immunohistochemistry (B3). The illustrations show granulocytic proliferation in an hypercellular bone marrow, with monocytes (CD14+), erythroid precursors and abundant hypolobulated and small megakaryocytes, suggestive of chronic myelomonocytic leukemia. The last image (B4) shows the detection by NGS (S5 Gene Studio) in both BM and peripheral blood of the same KRAS mutation already detected in the eyelid specimen. [file Image_1.jpg]
